# Hospital and Institutional News

**Published:** 1919-05-03

**Authors:** 


### HOSPITAL AND INSTITUTIONAL NEWS.

### INVALUABLE SERVICE THROUGH THE KING'S FUND.

His Majesty the King, patron of King Edward's Hospital Fund for London, has been pleased to appoint His Royal Highness the Prince of Wales President of the Fund, the appointment of His Royal Highness having been recommended by the Lord Chancellor, the Prime Minister, and the Governor of the Bank of England, in accordance with the Act of Parliament. His Royal Highness, the President, has appointed as members of the General Council of the Fund the Marquess of Cambridge, the Viscount Iveagh, and the Rt. Hon. James W. Lowther, M.P., Speaker of the House of Commons, who since 1910 have exercised as Governors the powers of President. The three Governors in question throughout the war have rendered invaluable service to the hospitals of London through their devotion to the interests of King Edward's Hospital Fund, and the untiring and able manner in which they have fulfilled the duties of Governors of the Fund. Points of great importance to the welfare and maintenance of the hospitals have arisen during the time they have held these important offices, and the wisdom they have exercised, and the whole-hearted service they have rendered throughout the period, have greatly contributed to the welfare and progress of the hospitals through a most trying time, now happily ended.

### THE HONOURS LIST.

Long deferred, the New Year Honours List of the Prime Minister was issued on Monday night. Among the recipients are the following:—To be a Baron: Sir Thomas Robert Dewar, Bt., J.P., D.L., President of St. Paul's Hospital, and worker on numerous hospital committees. To be a Baronet: Norman Moore, Esq., M.D., F.R.C.P., President of the Royal College of Physicians, Consulting Physician and Emeritus Lecturer on the Principles and Practice of Medicine at St. Bartholomew's Hospital and University of London, Representative of the Royal College of Surgeons on the General Medical Council. To be Knights: Alderman George Bean, Governor of Dudley Guest Hospital; Lieut.-Col. Joseph Montagu Cotterill, C.M.G., F.R.C.S., M.B., and C.M. (Edin.), R.A.M.C. (T.), Consulting and late Acting Surgeon, Edinburgh Royal Infirmary, and Lecturer in Clinical Surgery, Edinburgh School of Medicine, late President of the Royal College of Surgeons, Edinburgh, Commandant during the War of Craigleith Military Hospital; Arthur Lucas, Esq., for forty years Chairman of the Great Ormond Street Hospital for Children; Edward Malins, Esq., M.D., F.R.C.P., J.P. for the County of Warwick and the City of Birmingham; Thomas Jenner Verral, Esq., M.D., Chairman of the Central Medical War Committee for the past four years, greatly assisted the Army Council and the Ministry of National Service in obtaining medical officers for the Services; James Gadesden Wainwright, Esq., J.P., late Treasurer of St. Thomas's Hospital.

### CONSULTING SURGEON TO THE ARMY OF OCCUPATION.

Colonel Harold Burrows, M.B., B.S. (London), F.R.C.S. (Eng.), has been appointed Consulting Surgeon to the Army of Occupation. This is a position of great responsibility and importance which may offer a wide and important field for the exercise of gifts of a high order which have continuously won for Colonel Burrows the fullest recognition and appreciation wherever his work has lain, throughout a busy career of active surgical experience. His gifts and devotion to his profession marked him out at an early stage in his career as a man, who, with opportunities, was bound to gain a high place in his profession. The result has proved the justice of the anticipation, and his considerable talents and genial personality will make his appointment as Consulting Surgeon to the Army of Occupation welcomed by all ranks.

### THE FUTURE IN MESOPOTAMIA.

Of interest to the many medical men who have had experience of Mesopotamia during the past few years appears a letter in the *Times* from Dr. T. Barrett Heggs, late Medical Officer of Health of Bagdad, suggesting that a unique opportunity occurs for creating there a Health Service on the most modern lines. He points out that an essential superiority over those of more Eastern British possessions is found in the customs, ambitions, and intelligence of the Mesopotamian people, who are already appreciative of the introduction of many Western ideas. The population have the greatest faith in European medicine, sanitation and preventive administration, and Dr. Heggs, at the head of several suggestions of organisational details, emphasises that public health measures in these parts should be based on the European rather than the Indian model. Such a question relating to a land, the future of which is bound soon to come prominently under official consideration, appears to us of the greatest importance in the founding of a healthy and contented colony.

### DOCTORS IN UNION.

As we go to press we understand there is being held a Conference, the understood object of which is to bring together representatives of all professional organisations whose work bears upon questions of national health, so that the detailed constitution of a permanent Medical Parliamentary Committee may be at last decided. Undoubtedly the existing Committee has, considering its limitations as a representative body, made appreciable progress towards the solution of many difficult problems of these times, but the convening of this Conference and the bringing together of so many previously divergent branches of medical opinion constitutes the Committee's most valuable action. Prominent members of the hospital administrators, the pharmacists and nursing profession are invited, as well as representatives from every important medical association in the country are to take part. The resulting discussions will be watched with the greatest interest, not only as showing what form the new Committee will take, but as the first steps towards a real union of the doctors with both the administrative authorities and their own professional colleagues. It is a hopeless task to produce an ideal foundation for the health work in this country until this object is in a great measure achieved.

### POST-GRADUATE TRAINING.

A meeting of university, hospital, and medical school representatives was held on April 29 at the Royal Society of Medicine, and consideration given to the formation of a Post-Graduate Medical Association. Sir William Osler, who presided, outlined the objects and requirements of the new movement to which we have often previously referred in this Journal. In future in the London Undergraduate Medical Schools there will be annually two courses of post-graduate teaching of a fortnight to one month in length, the programme being drawn up three months in advance. The Council of the Association will consist of representatives from all teaching institutions and from the Ministry of Health, the Dominions, and the United States. The permanent secretarial staff will collect medical literature to be at the disposal of all medical men, and their offices are to form a permanent centre for information for all undertaking post-graduate work.

### AMERICAN CHILD WELFARE.

The American Department of Labour is planning a conference in Child Welfare, to be held in Washington about May 6, to which all qualified to speak regarding the War's lessons on the health, education, and work of children have been invited. It is hoped the meeting and discussion will be international in character and after the main conference, secondary district problems will be dealt with at convenient local centres throughout the months of May and June. Much valuable information and a wealth of new ideas may be forthcoming from this progressive nation, and those people in this country interested in children's problems may find much to interest them in the American literature of the near future.

### THE PLACE OF VOLUNTARY HOSPITALS.

A great many interesting points were made by Mr. E. W. Morris, House Governor of the London Hospital, at the Hospital Saturday Fund meeting at the Mansion House, and although his progressive views may not meet with general acceptance, they throw a bright light on many of the failures and possibilities of our present system. He suggested that in view of the sometimes enormous pressure of patients passing through them, the central hospitals of London should act as casualty clearing-stations and be linked by ambulance with a ring of convalescent homes like the convalescent camps in France. Also that a central bureau and organisation should be developed, where a medical man could ascertain by telephone where there was an available bed for his patient instead of the sufferer being zig-zigged in an ambulance all over London until a vacancy was found. Lack of co-ordination in hospitals' outlay, so that economy could not be effected by combined purchase and the incidental expense of individual testing of drugs, foods, materials, etc., was criticised. Ample provision of pay beds for people of sufficient means, a skilled and adequate number of the medical staff to be full-time and salaried, provision for post-graduate experience of older men even after years of private or government medical practice, and extensive revision in both the methods and curriculum of medical teaching were all, in the speaker's opinion, of urgent importance. He deplored the existing multiplicity of Boards and Government departments in control of medical administration. The voluntary hospitals now, wedged in among all these, stood in semi-isolation, and unwillingly formed part of the present chaotic arrangement for the treatment of the sick. His words truly make one feel there is much room for many reforms.

### HOSPITAL SATURDAY FUND RECORD.

Last year created a record for the Hospital Saturday Fund. The contributions amounted to £70,182, a sum which exceeded by £10,954 the income of the previous year, and the largest in the history of the Fund. A large advance was also recorded in the number of benefits granted, the total being 51,383, as compared with 46,491 in 1917. Awards were made as follows:—general, cottage, and special hospitals, £40,833; convalescent homes, £2,457; dispensaries, £382; nursing institutions, £842; distribution committees, £3,000; surgical appliances committee, £3,500; ambulance committee, £657; miscellaneous, £308. The total distributed was £52,579, compared with £45,431 twelve months ago. The sum of £4,815 went in management expenses, which represents 6.9 per cent. of the gross receipts.

### THE SECRETARYSHIP OF THE SATURDAY FUND.

We are sorry to learn that Mr. A. W. Davis has resigned the secretaryship of the London Hospital Saturday Fund, though his resignation will not take effect till mid-summer. After having served the Fund as organising secretary for twelve years, though this represents barely more than half of his active association with it, Mr. Davis finds that his eyesight needs closer care than he has been able of late to bestow upon it. Since he succeeded the late Mr. W. G. Bunn in October 1906, the Fund's work has so grown that he has often had to devote as much as ten or even twelve hours to its office. His record of regular work has never been interrupted by any breakdown in health, and he has amply earned his leisure. We hope that the Fund will adequately recognise by a generous pension the many years which Mr. Davis has devoted to its work, so that he may retire with as light a heart as the separation permits to a man of his keenness and energy. Mr. Davis has measured his time by the demands of the work only, and how heavy they have been we know. It is for the Hospital Saturday Fund to mete to him again his own generosity, that he and Mrs. Davis may enjoy their leisure as amply as the Fund has enjoyed the active years of his life. The Fund passed many a crisis under his guidance, and the memorial of his work is its growing influence and usefulness now that he is leaving the helm.

### THE WORK OF THE CHARITY COMMISSIONERS.

According to the sixty-sixth report of the Charity Commissioners for England and Wales, the value of new endowments settled in 1918 upon permanent trusts for investment amounted to £1,166,712, and, except in a few cases, this amount does not include real or personal property settled for charitable purposes by deed or gifts *inter vivos*. £426,368 of the sum quoted is in respect of medical charities. A large section of the report of the Commissioners deals with the administration of the War Charities Act, 1916. During 1918 the number of charities registered was 2,555; seven charities were refused registration, and one was removed from the register. The chief work now as regards war charities concerns the question of unexpended balances, and the duty of winding up charities has been referred to a body of trustees called "The Central Trustees of Controlled War Charities." The application of surplus funds of the British Red Cross Society and the Order of St. John of Jerusalem is regulated by a separate Act, framed in consultation with the Charity Commissioners. The experience of the working of the War Charities Act has confirmed the previously expressed view of the Commissioners that further legislation is desirable, and the question of extending similar provisions to all charities, for whatever purpose, is one which must be considered.

### MR. FRANCIS MOORE'S 33 YEARS' SERVICE.

At the time of Mr. Moore's appointment in 1886, plans for the rebuilding of the Royal Infirmary, Liverpool, had been prepared, and a commencement was made the following year. The new building was opened by the late Duke of Clarence in 1890, at a cost (with equipment) of £181,000, free of debt, this desirable end being mainly due to the admirable handling of the funds during the few years they were available by the then Treasurer, the increased value of the investments in which they had been placed, with the interest on them amounting to no less than £28,000. To meet the expected increased cost of maintainance in the new building, a "Maintenance Fund," which amounted to £25,000, was subscribed at the same time with the intention of spreading it over ten years, but it served its purpose for a much longer period. The attendances of out-patients increased so largely after the opening of the new building that it became necessary to make an extension of the hospital to cope adequately with this branch of the work, and in 1911 a new out-patient department was completed at a cost of £45,000; and another "Maintainance Fund" amounting to £17,000 was contributed. The great need at the moment is a new Nurses' Home; the present building adjoining the hospital being antiquated and not having sufficient accommodation for the whole staff, part of whom have to be housed in an adjoining street under conditions which are inadequate for proper comfort. For this purpose a sum of £10,000 has already been raised; but in view of the present cost of building, this amount must be largely added to before a commencement of this extension can be contemplated. During recent years the cost of maintainance has so largely increased that it has been necessary to use all untied Legacies and Donations for current expenses, but from the year 1886 until the present time the dividends from Investments have increased from £1,937 to £6,053—good evidence, taken in conjunction with the large sums above referred to, of the liberality of Liverpool citizens, and of the careful handling of the Funds by those responsible for the management of the hospital.

### CURATIVE WORKSHOPS AT NETLEY.

Curative workshops have just been added to the Netley Hospitals. They occupy a position on high ground between the Royal Victoria and British Red Cross Hospitals and form a range of bright, airy, and artistic buildings. Here, patients at the hospitals may receive, gratuitously, instruction in the selected of a large number of trades. Apart from the advantage of acquiring skill in a new branch of work if a man has been disabled for his old employment, there is in such training, curative aid to nerves and muscles, which is not the least of the objects aimed at. It may be well to dispel an erroneous impression which prevails that the ability thus acquired will in some way affect the pension due to a man. Such fears are groundless. The workshops are under the direction of Captain Hylton Lamotte, who, besides winning a reputation as motor specialist, has made a special study of surgical appliances. He was appointed by the War Office.

### AN UP-TO-DATE CHARITY.

For over one hundred years the City of London Truss Society has done exceptionally good work, and at the present time is called upon to do more than ever. It was founded in 1807 by Mr. John Taunton, M.R.C.S., grandfather of the present secretary, for the relief of the ruptured poor throughout the United Kingdom, and one of the most important features is that whatever the cost of the instrument may be—sometimes it is at least five guineas—the patient can obtain it, with the addition of surgical advice, with one letter. The War has brought changes, for there are many City Clerks suffering from hernia, brought on by service in the field, who find it almost impossible for them to pay for skilled surgical advice, and the full cost of the truss. At the same time they do not like to come to an institution like the City of London Trust Society *in formâ pauperis*. The Society has decided to meet these cases, the patient contributing towards the cost of treatment. Ever since its formation the Society has enjoyed Royal patronage, the King being its present patron.

### COTTAGE HOSPITAL AND RECONSTRUCTION.

In connection with the Woking War Memorial it is hoped to enlarge the Victoria Cottage Hospital, an institution much in request and already looked to in every emergency. Nothing could better show the value of cottage hospitals than the way in which they are taking part in the reconstruction of our hospital system. The casualties which it received last year were very numerous. The Red Cross Society has asked and received permission to build huts in the hospital's grounds, two of which will be for sailors and soldiers, and the third for civilian patients. The Surrey County Council has begged and received permission to use the out-patients' hall for venereal cases, and an $x$-ray department is likely to be added if proper support is obtained. The work also done by the hospital for the school care committee is another link between it and the town. Therefore, since Woking is perhaps worse off than other places for hospital beds, and already is so much indebted to the Victoria Cottage Hospital, there seems no reason why the enlargement of the hospital should not be the War memorial of the town. Mr. R. M. Driver, the hon. secretary, and Miss Stevens, the Matron, have had a heavy burden to bear. The public authorities appreciate such an invaluable ally as the hospital. It only remains for the townspeople to second the efforts being made on all sides for them.

### NEW SECRETARY TO LIVERPOOL ROYAL INFIRMARY.

Mr. William Rutter, Resident Secretary at the Chester Royal Infirmary, has been appointed General Superintendent and Secretary in place of Mr. F. H. Moore, who is retiring after 33 years service. Mr. Rutter, whose age is 31, received his early training in hospital work at the North Staffordshire Infirmary, Stoke-on-Trent, where he was engaged eight years, until appointed Assistant Secretary to King Edward VII's Hospital at Cardiff, in 1903. This latter post he held for twelve months before occupying his present position at Chester, from which he has been absent three years with the Military Forces during the War, in which he became Captain and Adjutant of a Machine-Gun Battalion, and was mentioned in despatches.

### AN IRISH DILEMMA.

The Benn Ulster Eye, Ear, and Throat Hospital is a remarkable Irish hospital, as the following facts show. Built by Mr. Benn in 1873 at his own expense, and with a reputation in Belfast since the late Dr. McKeown began his good work there, Dr. Killen's investigations seem to show that 80 per cent. of the income is provided by the patients themselves, and the remainder mainly from investments, with a few subscriptions thrown in. No wonder the Lord Mayor complains of public apathy. It is an extreme case. But the hospital's friends, from Sir John Byers downwards, show their belief in its work by actively supporting the new appeal for its reconstruction. The latter declares, and the point has been proved before now, that not only the work, and the staff, need new quarters, but that the whole "tone of the institution" would be raised if it were reconstructed properly. A certain London hospital could get no heed paid to its work till it was properly housed, nay, properly fronted. The public needs to be told what it ought to admire, and what to support, and there is a note of reality in this appeal which cannot fail to be rewarded.

### SHEFFIELD AND CO-ORDINATION.

The joint appeal for £100,000 which has been issued on behalf of the hospitals of Sheffield seems likely to develop into an attempt at co-ordination, more or less upon the Birmingham plan. Eight-tenths of the sum have already been raised. Mr. W. Irons, in a recent speech on behalf of the Royal Hospital, while alive to the value of further co-operation between the Sheffield institutions, expressed his belief in the voluntary principle, and warned his hearers that Government control would destroy the personal interest which is the human salt in voluntary work. Mr. J. H. Doncaster, the honorary treasurer, hopes that the joint appeal will lead to joint work in several directions. As we have often repeated, this achieved locally will further hospitals which combine to secure it in a strong position to check undue interference from the Minister of Health when he comes.

### COD-LIVER OIL PREPARATIONS.

The Medical Officer of Health for the City of London (Dr. Howarth, C.B.E.) has drawn the attention of the Corporation to the necessity for some standard being established for preparations in which cod-liver oil is an ingredient. Samples of so-called "extract of malt and cod-liver oil" contained from 6.3 to 15.4 per cent. of the latter, instead of the proper percentage of 33, while other preparations contained from 24.1 to 48.1 per cent. of cod-liver oil. Such variety, the Medical Officer thinks, is very confusing and unsatisfactory.

### THIS WEEK'S DRUG MARKET.

The market has begun to recover from the Easter holidays, but business has been practically at a standstill for more than a week, during which time, price changes of importance were conspicuous by their absence. Pending the Budget, speedy operations were especially cautious, and a revival of business was not to be expected before the Chancellor's pronouncement, while we cannot expect the markets to become fully active until the declaration of peace. In the meantime the general downward tendency of prices continues, although menthol, camphor, and peppermint oil are still to be noted among the exceptions. The prices of bromides are firmly maintained and a recession is not looked for in this market. For the most part, the downward tendency in the prices of synthetic drugs is still in evidence, and aspirin, salicylic acid, hexamine, paraldehyde, antipyrin, and resorcin are all somewhat cheaper. The considerable stocks of eucalyptus oil coupled with the smaller demand have resulted in lower quotations for this article.

